# Folate-Targeted Curcumin-Encapsulated Micellar Nanosystem for Chemotherapy and Curcumin-Mediated Photodynamic Therapy

**DOI:** 10.3390/polym12102280

**Published:** 2020-10-04

**Authors:** Yun Hsuan Lin, Ching-Yi Chen

**Affiliations:** Department of Chemical Engineering, College of Engineering, National Chung Cheng University, Chia-Yi County 621301, Taiwan; gdgd60329@gmail.com

**Keywords:** curcumin, photodynamic therapy, polymeric micelle, targeted drug delivery system

## Abstract

Curcumin (CUR) is a natural phenolic product used as a high-efficiency and low-toxicity anticancer drug and photosensitizer. However, it has a poor aqueous solubility and a lack of target specificity, which limits its clinical applications. Hence, we developed a folate-conjugated polymeric micelle to enhance the efficient delivery of CUR for effective cancer cell targeting and anticancer efficiency. A series of biocompatible folate-conjugated poly(2-(methacryloyloxy)ethylphosphoryl- choline)-*b*-poly(ε-caprolactone) (FPM) was synthesized with different hydrophobic lengths and folate contents. The prepared CUR-loaded micelles (CUR-FPM) possessed several superior properties, including an excellent drug loading capacity (6.3 ± 1.2%), improved CUR aqueous stability, fast-sustained CUR release in an acidic environment, and efficient intracellular production of reactive oxygen species. The in vitro cytotoxicity demonstrated that the CUR-FPM micelles efficiently suppressed the growth of HeLa cells (folate-receptor overexpression) compared to that of HT-29 cells, and a competition study showed less cytotoxic effect when free folic acid blocked the folate receptor, indicating the folate conjugation played the role of targeting the specific cells well. Moreover, the CUR-mediated photodynamic therapy (PDT) by CUR-FPM micelles under irradiation further inhibited the proliferation of cancer cells. All these results indicate that the CUR-FPM micelles could be a promising delivery system for folate-overexpressing cancer cells, complementary chemotherapy, and CUR-mediated photodynamic therapy.

## 1. Introduction

Curcumin (CUR) is a well-known natural hydrophobic phenolic compound extracted from the rhizomes of turmeric (*Curcuma longa*) and has been widely used in the treatment of a variety of diseases, such as skin diseases, wounds, liver disorders, and pulmonary and gastrointestinal systems [1]. It has been found that curcumin has antioxidant, anti-inflammatory, and antimicrobial activities and antitumor effects [2,3,4,5]. Several studies have confirmed that curcumin can not only effectively affect many growth factor receptors and cell adhesion molecules involved in tumor growth, angiogenesis, and metastasis, but also provide anti-inflammatory and antitumor effects by inhibiting the activity of transcription factor NF-kB and downstream gene products, including Bcl-2, COX-2, MMP-9, TNF-α, etc. [6,7]. In addition, Phase I clinical trials have shown the safety of curcumin at doses of up to 12 g per day [8]. However, its low water solubility, poor bioavailability, and rapid metabolism by the human body are essential factors that seriously limit its effective applications in the treatment of cancer and other diseases. To improve the above issues, various delivery nanoparticles, nanocomplexes, and conjugates have been developed for the encapsulation, transport, and delivery of CUR [9,10,11,12,13]. The results of these studies indicate that these new delivery systems enhance the water solubility and antioxidant and anticancer therapeutic efficiency of CUR. Nonetheless, safer, more effective, more optimally bioavailable, and easier to tailor-make delivery vehicles are still demanded for the delivery of curcumin in different application systems and administration routes.

In recent years, CUR has been found to be a potential photosensitizer, with a low toxicity and high efficiency [14,15] in photodynamic therapy (PDT) for the treatment of cancer and antimicrobial infection. PDT is recognized as a minimally invasive and effective therapeutic modality, with a better selectivity to targets that are exposed to light and fewer side effects, which renders it advantageous compared to conventional therapies. It is based on the simultaneous presence of a photosensitizer, light, and oxygen in the target tissue. When the photosensitizer is exposed to an appropriate wavelength in the presence of molecular oxygen, it produces reactive oxygen species (ROS)—e.g., hydroxyl radical and peroxide (type I PDT) or singlet oxygen (^1^O_2_, type II PDT)—that cause damage to nearby cells through apoptotic or necrotic mechanisms [16,17]. However, many photosensitizers, such as porphyrin-based molecules or curcumin, have a poor aqueous solubility that might cause the photosensitizers to aggregate in the physiological environment, resulting in them losing their photodynamic effect. Moreover, the dark toxicity and lack of target specificity of photosensitizers also limit their clinical treatment.

A phosphorylcholine (PC)-based polymer—e.g., poly(2-methacryloyloxyethyl phosphorylcholine) (PMPC)—with a zwitterionic structure has strong hydration and can effectively suppress non-specific protein adsorption and foreign body reactions [18]. Its unique properties mean that it holds promise as an alternative to conventional poly(ethylene glycol), which might have some drawbacks such as reduced cellular uptake, oxidation damage, and accelerated blood clearance. Although PMPC has been approved by the FDA for clinical use [19], drug carriers based on PMPC polymers still need to be developed and evaluated for different application systems in vitro and in vivo.

In this study, a series of folate-conjugated poly(2-methacryloyl-oxyethyl phosphoryl-choline)-*b*- poly(ε-caprolactone) (FA-PMPC-*b*-PCL, FPM) was developed to encapsulate CUR for the evaluation of their selective targeting and photodynamic anticancer effects compared to non-photoactive CUR. Two different FPM-block copolymers (FPM1 and FPM2) were synthesized with different hydrophobic PCL lengths and folate contents. The potential of this FPM micelle to encapsulate CUR so as to overcome the above-mentioned limitations has been evaluated by its photophysical properties, stability, size distribution, and morphology. The photodynamic effect of CUR-loaded micelles (CUR-FPM) was also evaluated. The cytotoxic, phototoxic effects, and specific targeting characteristics of CUR-FPM micelles against HeLa cells (folate receptor overexpression) and HT-29 (lack of overexpressed folate receptor) were investigated in vitro with/without light irradiation. The advantages of the CUR-FPM micelles for specific targeting and PDT are discussed. 

## 2. Materials and Methods 

### 2.1. Materials

2,2’-bipyridine (bpy, 98%, Alfa Aesar, Tewksbury, MA, USA), curcumin (CUR, >98%, TCI), 2,7-dichlorofluorescein- diacetate (DCFH-DA, Sigma-Aldrich, St. Louis, MO, USA), folic acid (FA, TCI, Tokyo, Japan), 2-methacryloyloxyethyl phosphoryl- choline (MPC, Sigma Aldrich, St. Louis, MO, USA), 1,1,4,7,7-pentamethyldiethylene- triamine (PMDETA, Acros, Fair Lawn, NJ, USA), and tin(II) 2-ethylhexanoate (Aldrich, Milwaukee, WI, USA) were used as received. ε-caprolactone was distilled over CaH_2_ under reduced pressure. Copper bromide (CuBr, >99.998%, Alfa Aesar, Tewksbury, MA, USA) was washed with acetic acid and ether and then dried under vacuum. All the solvents were purchased from J. T. Baker, ECHO, or Macron and used as received unless otherwise noted. Singlet oxygen sensor green (SOSG, Life Technologies, Carlsbad, CA, USA) was used according to the manufacturer’s instructions. 3-(4,5-dimethyl thiazol-2-yl)-2,5-diphenyl- tetrazolium bromide (MTT) assay was obtained from Bio Basic (Markham, ON, Canada). Minimum Essential Medium (MEM 1X) was purchased from Corning (Corning, NY, USA). Phosphate-buffered saline (PBS, 1X), amino acid (100X), sodium pyruvate (100 mM), and penicillin-streptomycin solution (100X) were commercially available from Bio West (Nuaillé, France). Hydroxyethyl 2-bromoisobutyrate (HEBiB) and propargyl folate was prepared according to the published protocols [20,21]. Poly(ε-caprolactone) (PCL-Br) macroinitiator (M_nNMR_ = 3631 g/mol, PDI = 1.25 and M_nNMR_ = 9331 g/mol, PDI = 1.30) was synthesized according to our previous study [22].

### 2.2. Synthesis of PMPC-b-PCL (PM) and N_3_-PMPC-b-PCL (N_3_-PM)

PMPC-*b*-PCL was prepared by atom transfer radical polymerization (ATRP). Typically, PCL-Br macroinitiator (461 mg, 0.13 mmol, M_nNMR_ = 3631 g/mol, PDI = 1.25) and CuBr (18.2 mg, 0.13 mmol) were added into a Schlenk tube, then vacuumed for 20 min. Under nitrogen atmosphere, a solution of MPC (1.5 g, 5.08 mmol) and bpy (39.7 mg, 0.25 mmol) in 3 mL of DMSO/MeOH mixture (v/v = 1/1) was bubbled with nitrogen for 20 min and transferred into the Schlenk tube, followed by a freeze–pump–thaw process three times. After stirring at an ambient temperature for 20 min, the solution was polymerized under nitrogen at 65 °C overnight. The reaction mixture was dialyzed (Cellu-Sep, MWCO 3500 Da) against MeOH for 3 days to remove the unreacted reactants and catalysis completely. The medium was replaced every 6 h. Finally, we evaporated the solvent, precipitated it into diethyl ether, and dried it in a vacuum oven to obtain PM1 copolymer (1.4 g, 73%). M_nNMR_ = 13965, DP_PCL_ = 30, DP_PMPC_ = 35; PM2 (2.2 g, 83%): M_nNMR_ = 18779, DP_PCL_ = 80, DP_PMPC_ = 32. 

For the synthesis of N_3_-PMPC-b-PCL, PMPC-b-PCL (PM1) (1.0 g, 0.07 mmol) was dissolved in 3.5 mL of DMF and 1.5 mL of MeOH mixture under nitrogen. Sodium azide (23.3 mg, 0.36 mmol) was then added to the mixture and reacted at 60 °C for 24 h. The reaction mixture was dialyzed (MWCO 3500) against DI water/MeOH mixture (1:1, v/v) for 24 h. The medium was replaced every 6 h. The product was precipitated by diethyl ether and dried in vacuum oven to obtain a product of around 863 mg (86% yield).

### 2.3. Synthesis of FA-PMPC-b-PCL (FPM)

Propargyl folate was prepared following the methodology described in the literatures [21,23]. The detailed synthetic procedures and characterization results are given in the Supplementary Information. Typically for the synthesis of FPM1, propargyl folate (37 mg, 0.08 mmol) and CuBr (6.2 mg, 0.04 mmol) were added into a Schlenk tube and vacuumed for 20 min. A solution of N_3_-PMPC-*b*-PCL (0.7 g, 0.05 mmol) and PMDETA (7.4 mg, 0.04 mmol) in a cosolvent of DMF/MeOH (3.5 mL/0.7 mL) was bubbled with nitrogen for 20 min and transferred into the Schlenk tube under nitrogen, followed by the freeze–pump–thaw process. Then, the reaction was operated at 65°C under nitrogen for 48 h. After the reaction, the mixture was transferred into a dialysis membrane (Cellu-Sep, MWCO 3500 Da) against deionized water for 1 day and changed to MeOH for 3 days. The medium was replaced every 6 h. The purified polymer was recovered by solvent evaporation, precipitated into diethyl ether, and dried under in a vacuum oven to obtain FPM1.

### 2.4. General Characterization

The sizes and zeta potentials of the blank or CUR-loaded micelles were determined by Malvern Zetasizer Nano-ZS90 dynamic light scattering (DLS, Malvern, Worcestershire, UK) equipped with a 633 nm He-Ne laser source at a 90° scattering angle. The measurements were performed at 25 °C with 1 mg/mL of micellar solution. Each sample was analyzed in triplicate. Micellar morphologies were performed by transmission electron microscopy (TEM, JEOL JEM-2010, Tokyo, Japan) at an accelerating voltage of 120 kV. The samples were prepared by dropping 20 μL of 1 mg/mL micelle suspension on the copper grid and dried at 40 °C for 1 h without any stain before measurement. The transformation of the bromine end group of the PM copolymers to the azide group was confirmed by Fourier transfer infrared (FTIR, Bruker Vertex 70v, Rheinstetten, Germany). Dried samples were pressed with potassium bromide (KBr) powder into pellets. The quantitation of folic acid on the FPM copolymers was carried out by a UV-visible spectrometer (Varian Cary 50, Walnut Creek, CA, USA). The aqueous stabilities of the CUR-loaded micelles and free CUR were investigated by monitoring the change in CUR absorbance in a pH 7.4 buffer solution over a period of 24 h at 37 °C using a UV-visible spectrometer. At a predetermined time, 200 μL of the CUR-loaded micelles or free CUR solution was drawn and diluted with 1.5 mL of DMSO/MeOH/THF (equal volume) mixture. The solution was recorded by a UV-visible spectrometer. The average normalized absorbance from the triplicate experiments was plotted as a function of time. Free CUR was investigated with the same approach as the control group.

### 2.5. Preparation of Blank and CUR-Loaded Micelles

For the blank and CUR-loaded micelles (CUR-FPM), a solution of FPM copolymers (3 mg/mL) was mixed with different weight of CUR (CUR/FPM weight ratios: 0%, 3.33%, 5%, and 10%) in mixed solvent (DMSO: MeOH: THF = 1:1:1, v/v/v). Then, the mixture was injected slowly (25 μL/min) into ultrapure water to give the final polymer concentration of 1 mg/mL. After injecting, the mixture was dialyzed against ultrapure water or Britton–Robinson buffer solution (B–R buffer solution) of different pH values using a dialysis membrane (MWCO 3500 Da) to remove the solvent or unloaded CUR for another 24 h. The ultrapure water or buffer solution was replaced every 6 h. Finally, the micellar solutions were filtered through a 0.45 μm micro-filter, lyophilized, and stored at 4 °C. Weighted amounts of the lyophilized CUR-loaded micelles were dissolved in the mixed solvent to determine the drug loading capacity (LC) and drug encapsulation efficiency (EE). The EE and LC in each micellar type were quantified using the UV-visible spectra with reference to a calibration curve of CUR in mixed solvent. The LC was calculated from the ratio of the weight of drug loaded in micelles to the total weight of the micelles. The EE was calculated from the ratio of the weight of drug loaded in the micelles to the total weight of drug fed.

### 2.6. Determination of Release Kinetics of CUR

The CUR release profiles were studied by the dialysis method at 37 °C in pH 7.4 and pH 5 buffer solution (B–R buffer solution), respectively. Briefly, two sets of 3 mL of CUR-loaded micelles (CUR-FPM, 1 mg/mL) in dialysis membrane (MWCO 3500 Da) were immersed in 60 mL of different pH buffer solutions, respectively, and put in a shaking water bath at 37 °C. At specific time intervals, 100 μL of the micellar solution was drawn and further diluted with mixed solvent (DMSO: MeOH: THF = 1:1:1, v/v/v). The amount of CUR was quantified by absorbance measurement, with reference to a calibration curve of CUR in mixed solvent. The release percentages were calculated based on the absorbance changes.

### 2.7. Intracellular ROS Detection

The intracellular ROS generation was measured using the DCFH-DA staining method. HeLa cells were cultured in a 24-well plate at a density of 5 × 10^5^ cells per well for 24 h and incubated with 500 μL of DCFH-DA working solution for 30 min, followed by washing with PBS to remove the excess DCFH-DA. Then, 500 μL of free CUR or CUR-FPM micelles with a CUR concentration of 10 μg/mL was added and incubated for 2 h. After rinsing with PBS, the cells were immediately irradiated with a halogen lamp (0.04 W/cm^2^) for 10 or 20 min. The fluorescent images of the reaction product 2,7-dichlorofluorescein (DCF) were monitored using the confocal laser scanning microscope (CLSM, Olympus FV1000, Tokyo, Japan) for excitation at 473 nm and a bandpass emission filter set to 510–610 nm. The average DCF fluorescence intensities were analyzed with the ImageJ software over the CLSM images of each sample.

### 2.8. Cell Culture and Cell Viability

HeLa (human cervical cancer cell line) and HT-29 (human colon adenocarcinoma cell line) cells were seeded in the 96-well plate at a density of 6 × 10^3^ cells per well and incubated at 37 °C in a 5% CO_2_ atmosphere for 24 h. The cancer cell line was cultured in minimum essential medium supplemented with 10% fetal bovine serum and 1% antibiotics (antibiotic–antimycotic solution), amino acid, and sodium pyruvate. Then, the cells were treated with different concentrations of blank micelles, free CUR, and CUR-FPM micelles. After 24 h, the cells were washed twice with PBS and added to fresh medium containing MTT solution for incubation for another 3 h. Subsequently, the culture medium was removed and 50 μL of DMSO was added to each well to dissolve the internalized purple formazan crystals. The absorbance was measured at a wavelength of 570 nm using a microplate spectrophotometer (BioTek UQuant MQX200, Winooski, VT, USA). In the free folate competition study, 1 mM of folic acid and CUR-FPM micelles were added simultaneously to the HeLa cell incubation medium for 24 h. After washing, the cell viability was evaluated with an MTT assay. Each experiment was conducted in four replicates per plate. Two plates were used. The results were expressed as the percentage of the absorbance of the blank control.

### 2.9. In Vitro Phototoxicity Study

HeLa cells were incubated with free CUR and CUR-FPM micelles with a CUR concentration of 10 μg/mL for 2 h. Then, the cells were washed twice with PBS and immediately irradiated with a halogen lamp (0.04 W/cm^2^) for different periods of time. After that, the plates were incubated for another 24 h. The cell viability was evaluated with an MTT assay. Each experiment was conducted in three replicates per plate. Two plates were used.

### 2.10. Subcellular Localization Study

The subcellular localization of the free CUR and CUR-FPM micelles with/without free folic acid were investigated with confocal laser scanning microscopy (Olympus FV1000, Tokyo, Japan). HeLa cells were grown on the coverslip in 24-well plate at a density of 3 × 10^5^ cells per well and cultured at 37 °C in a 5% CO_2_ atmosphere for 24 h. The cells were treated with free CUR, CUR-FPM1, and CUR-FPM2 with/without 1 mM of free folic acid at an equivalent CUR concentration of 10 μg/mL for 2 h. Then, all the cells were washed thoroughly with PBS and fixed with 4% paraformaldehyde for 10 min, and successively stained with DAPI (10 μg/mL) for 15 min. After the cells were rinsed with PBS, the cells were mounted on a slide for fluorescent detection by CLSM. The fluorescence was obtained with the excitation wavelengths of 405 nm for DAPI and 488 nm for curcumin.

### 2.11. Cellular Uptake Study

The cell uptake study was quantitatively analyzed according to the reported protocol [10]. HeLa cells were seeded in 24-well plate at a density of 3 × 10^5^ cells per well and cultured at 37 °C in a 5% CO_2_ atmosphere for 24 h. The cells were then treated with free CUR, CUR-FPM1, and CUR-FPM2 with/without 1 mM of free folic acid at the CUR concentration of 10 μg/mL. After incubation of 2 h, the cells were washed with PBS, trypsinized, and resuspended in fresh MEM culture medium. The cells were centrifuged at 1000 rpm for 5 min at 4 °C. The resulting cell pellet was further mixed with 200 μL of methanol and sonicated to extract CUR from the treated cells. The cell lysate was centrifuged at 13,000 rpm for 10 min at 4 °C. The absorbance of the CUR was recorded at a wavelength of 450 nm using a microplate spectrophotometer. The amount of CUR was quantified on the basis of a calibration curve of CUR in methanol.

## 3. Results and Discussion

### 3.1. Preparation and Characterization of FPM

**Scheme 1:**
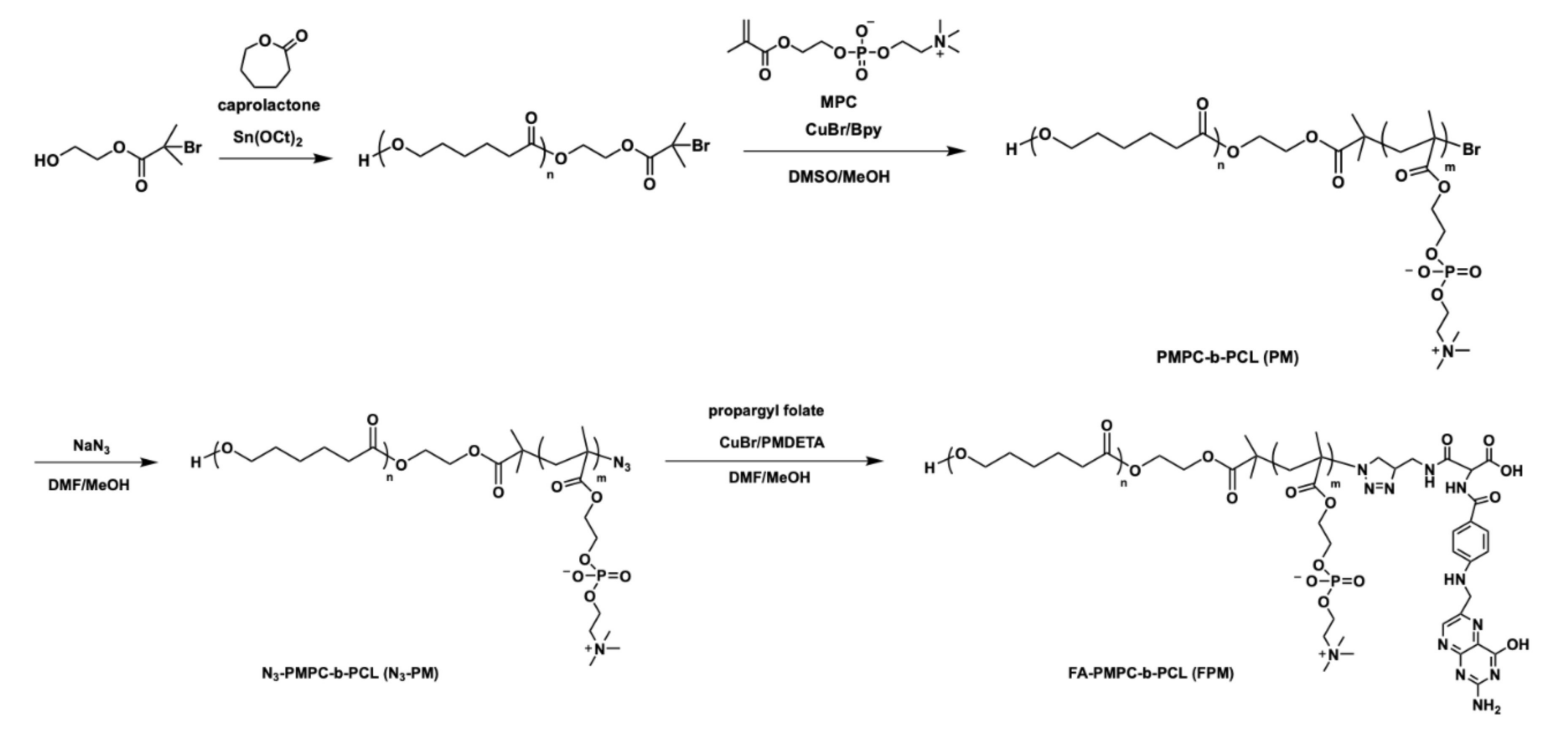
Synthetic route of the FA-PMPC-b-PCL (FPM) copolymer.

In this study, a series of folate-conjugated poly(2-methacryloyl-oxyethyl phosphorylcholine)-*b*- poly(ε-caprolactone) (FA-PMPC-*b*-PCL, FPM) was prepared via ring-opening polymerization (ROP), atom transfer radical polymerization (ATRP), and click reaction according to our previous synthetic procedures and published protocols [22,23,24]. The synthetic route is shown in Scheme 1 and the polymer characterizations are given in the Supplementary Material (Table S1 and Figures S1–S3). Two different amphiphilic block copolymers (FPM1 and FPM2) were synthesized with different hydrophobic PCL lengths and folate contents to investigate their effects on the properties of CUR, specific targeting characteristics, and anticancer efficiency. The purity and compositions of the synthesized FPM polymers were fully characterized by the ^1^H NMR spectra, GPC, and FTIR, and are summarized in Table S1. The coupling of propargyl folate onto the azide-terminated PMPC-*b*-PCL was confirmed by the complete disappearance of the peak of the azido group at 2093 cm^−1^ and the appearance of the characteristic peaks of folic acid at 1605 cm^−1^, which was attributed to the stretching vibrations of the aromatic C=C bond (Figure S3). This suggested a successful click reaction between the azido and alkyne groups. The quantitative coupling of folate onto azide-terminal PM was determined by UV–visible spectrometer with the folate calibration curve. The folate content of the FPM1 and FPM2 were 12 and 22 mg/g of polymer, respectively. 

Size is a key factor for polymer micelles because it has a significant impact on the in vivo performance of drug carriers. An appropriate size can ensure a low level of non-specific uptake by the mononuclear phagocyte system (MPS), minimal renal excretion, and the promotion of enhanced permeability and retention (EPR) effects to achieve the passive targeting of tumors [25]. Because of the amphiphilic nature of the FPM copolymers, FPM1 and FPM2 can self-assemble into micelles in aqueous medium. The average hydrodynamic diameters of the FPM1 and FPM2 micelles measured by DLS were around 162.7 ± 11.4 and 192.7 ± 14.5 nm with the narrow unimodal distribution, respectively. The larger micelle size of FPM2 than FPM1 was due to the longer hydrophobic PCL segment of FPM2, which formed the larger core of the micelles. The negative zeta potential of the FPM micelles was possibly due to the deprotonation of α-carboxylic acid of folic acid in a neutral environment (the reported pK_a_ of α-carboxylic acid is 2.5) [26]. TEM images showed the presence of well-dispersed spherical FPM micelles, with average diameters of around 159 ± 40 and 172 ± 40 nm for both FPM1 and FPM2 (Table 1 and Figure S4), respectively. The sizes were slightly smaller than those obtained using the DLS measurement. This might be attributed to the shrinkage of the polymer chains in the dried/dehydrated state. The critical micelle concentrations (CMCs) of the FPM micelles were determined by a fluorescence probe technique using pyrene for probing the polarity of its microenvironment. The CMCs were found to be 2.5 × 10^−3^ and 6.0 × 10^−4^ mg/mL in aqueous solution for FPM1 and FPM2, respectively. The decrease in the CMC value with an increase in the length of the PCL segment is agreement with the increment in the hydrophobic−hydrophobic interaction.

### 3.2. CUR Encapsulation and Aqueous Stability in Micelles

**Figure 1:**
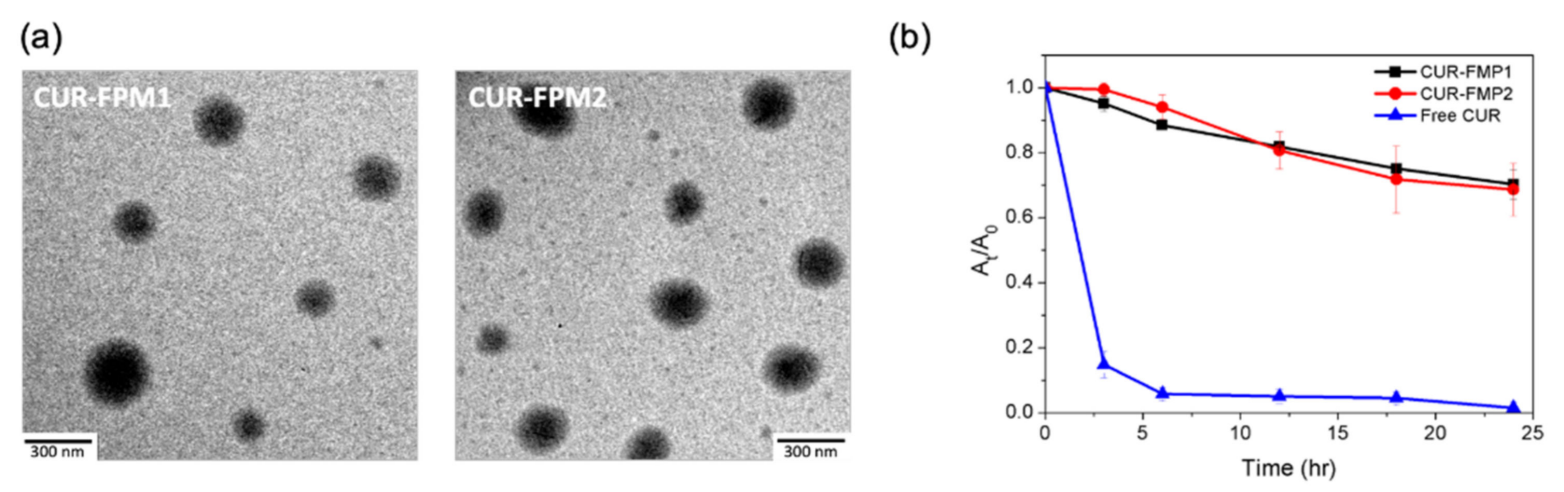
(**a**) TEM images of CUR-FPM micelles; (**b**) aqueous stability of free CUR and CUR-FPM micelles in PBS (pH 7.4) at 37 °C. A_0_ and A_t_ are the absorbance of CUR at 428 nm initially and time t, respectively.

FPM polymers were employed to encapsulate CUR and the prepared CUR-FPM micelles were lyophilized and stored at 4 °C. The drug encapsulation efficiency (EE%), drug loading capacity (LC%), and particle sizes of the CUR-FPM micelles are summarized in Table 1. The drug loading capacity showed the increment with increasing the drug to polymer feeding ratios, but the drug encapsulation efficiency showed that the FPM micellar core has the maximum limit loading capacity at a drug/polymer weight ratio of 5% (Figure S5). The FPM micelles showed a higher LC and EE compared to other PCL-based micelles [27], but had similar or less EE than other folate-conjugated PLGA- [28] or PLA-based [29] polymeric micelles and liposomes [30,31]. TEM images confirmed the increased size of the CUR-FPM micelles compared to the blank ones, but the spherical shape was unaltered after drug encapsulation (Figure 1a).

To study the aqueous instability of CUR, the CUR-FPM micelles and free CUR were studied in PBS (pH 7.4) at 37 °C, and we monitored the change in the CUR concentration using a UV-visible spectrometer over 24 h (Figure 1b). The free CUR showed a rapid degradation to less than 6% of its original concentration within 6 h, indicating the severe aqueous instability of free CUR. However, both CUR-FPM1 and CUR-FPM2 micelles showed an improved CUR aqueous stability at the same condition. The CUR concentrations still remained above 89% and 70% after 6 and 24 h, respectively, indicating that the FPM micelles significantly enhanced the stability of CUR by protecting the encapsulated CUR against hydrolysis and biodegradation.

### 3.3. In Vitro Release Behavior

**Figure 2:**
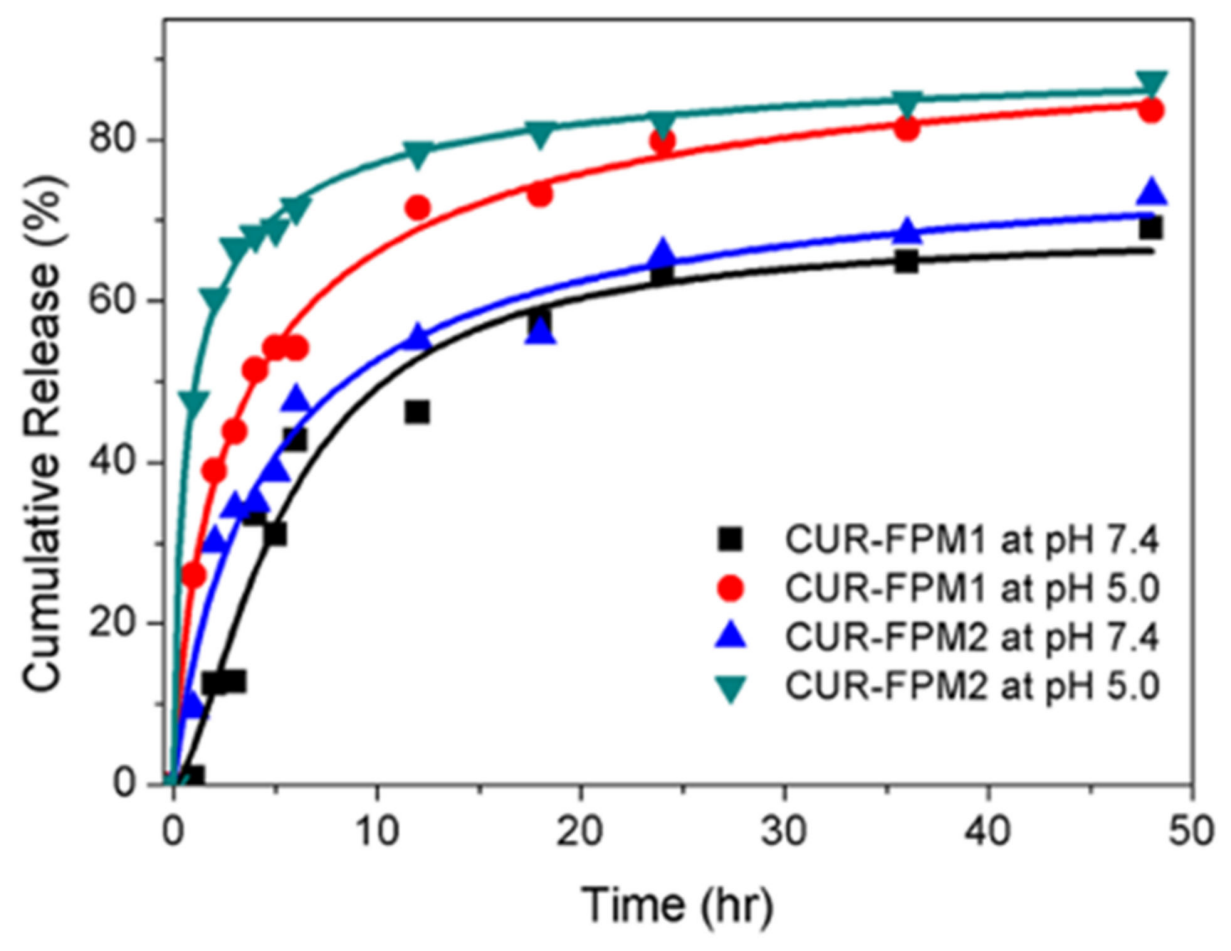
Cumulative CUR release from the CUR-FPM1 and CUR-FPM2 micelles at different pH values.

The drug release behavior of CUR-loaded micelles was investigated in pH 7.4 and pH 5.0 buffer solution at 37 °C to imitate the condition of blood circulation and the intracellular condition of cancer cells, respectively. The released amount of CUR was quantified by absorbance measurement with reference to a calibration curve of CUR in mixed solvent (Figure S6). As shown in Figure 2, a faster release rate of about 85% of CUR released from micelles after 48 h was observed at pH 5.0 than that at about 66% of CUR released at pH 7.4. This might be attributed to the faster degradation of the PCL core at a lower pH. In addition, the salts in the buffer solution might affect the conformation of the PMPC block and further lead to the expansion of the micellar core through ionic interaction [18,32], which also promotes drug release. An initial burst release was observed for the CUR-FPM2 micelles. This might be due to the drugs being located in the shell or between the shell and the core. The lengths of the PCL segments showed no obvious effect on the release rate at the same pH condition.

### 3.4. Intracellular ROS Generation Monitoring

**Figure 3:**
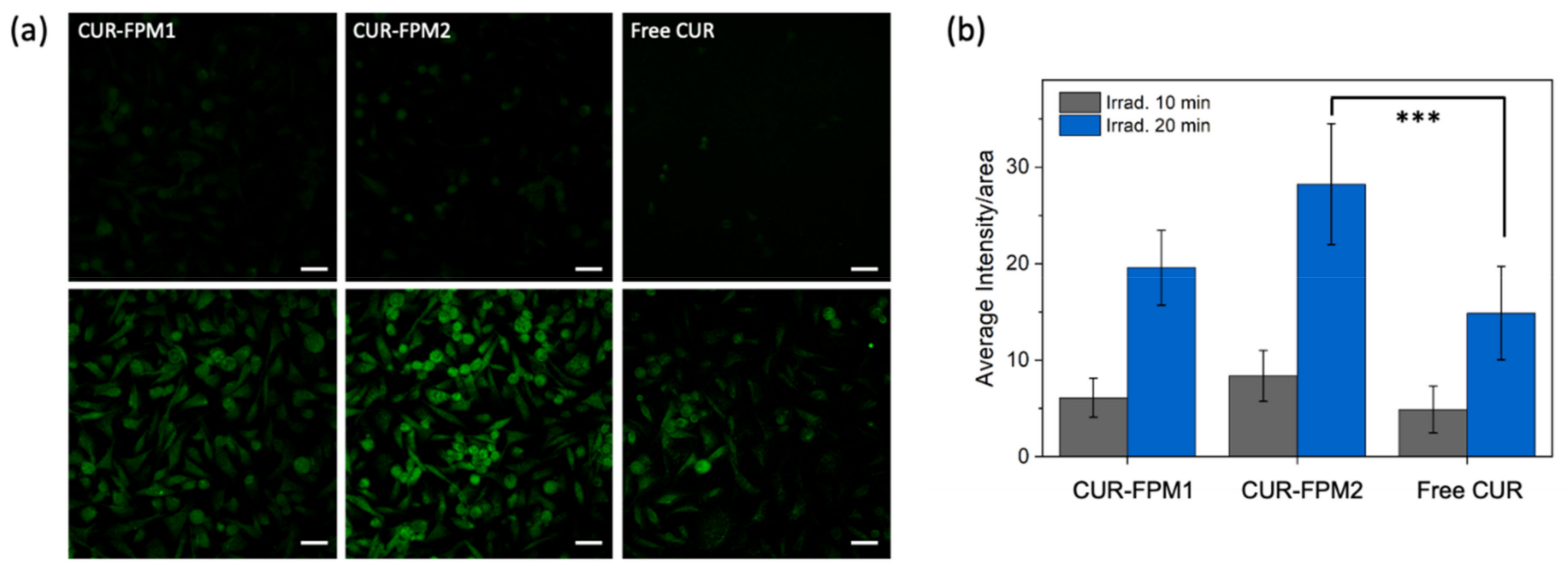
(**a**) CLSM images of HeLa cells treated with CUR-FPM1, CUR-FPM2 micelles, and free CUR under light irradiation (0.04 W/cm^2^) for ROS detection using DCFH-DA. Top row: irradiation for 10 min. Bottom row: irradiation for 20 min. Scale bar: 25 μm. (**b**) Average DCF fluorescence intensity per area of the CLSM images in each sample, analyzed by the ImageJ software. Data are presented as mean ± standard deviation. *p* values: *** *p* < 0.001, significance level.

The production of reactive oxygen species (ROS) is the most essential factor in PDT against cancer cells. To investigate the intracellular production of ROS induced by CUR-FPM micelles upon light irradiation, a cell-permeable ROS-detecting fluorescent probe DCFH-DA was used. It can easily enter into cells and be deacetylated by cellular esterase to form a non-fluorescent 2,7-dichlorodihyrofluorescein diacetate (DCFH), and then be oxidized by ROS to turn to highly fluorescent 2,7-dichlorofluorescein (DCF). In this study, CUR-loaded micelles (CUR-FPM1 and CUR-FPM2) and free CUR at an equivalent CUR concentration of 10 μg/mL were incubated individually with HeLa cells for 2 h and then irradiated with light (0.04 W/cm^2^) for 10 or 20 min in the presence of the ROS-detecting probe. Control experiments were conducted under the same conditions, but without light irradiation. There was no DCF fluorescence observed in the control groups (data not shown). The ImageJ software was used to analyze the DCF fluorescent signals to calculate the average fluorescence intensity per unit area of each sample. As shown in Figure 3, a stronger green fluorescence of DCF was observed in the CUR-FPM micelles as compared to that of free CUR. This implied the efficient delivery of CUR by FMP micelles and enhanced ROS production inside cells upon irradiation. In contrast, the weak fluorescence of free CUR might be due to the aqueous instability of CUR, which was consistent with the results of the CUR stability. Moreover, the ROS production within HeLa cells also remarkably increased in a light–dose dependent manner. The higher ROS production of CUR-FPM2 than CUR-FPM1 might be due to the increased folate conjugation of FPM2, which facilitated the endocytosis ability of micelles and further induced greater intracellular ROS generation.

### 3.5. In Vitro Cytotoxicity Evaluation

**Figure 4:**
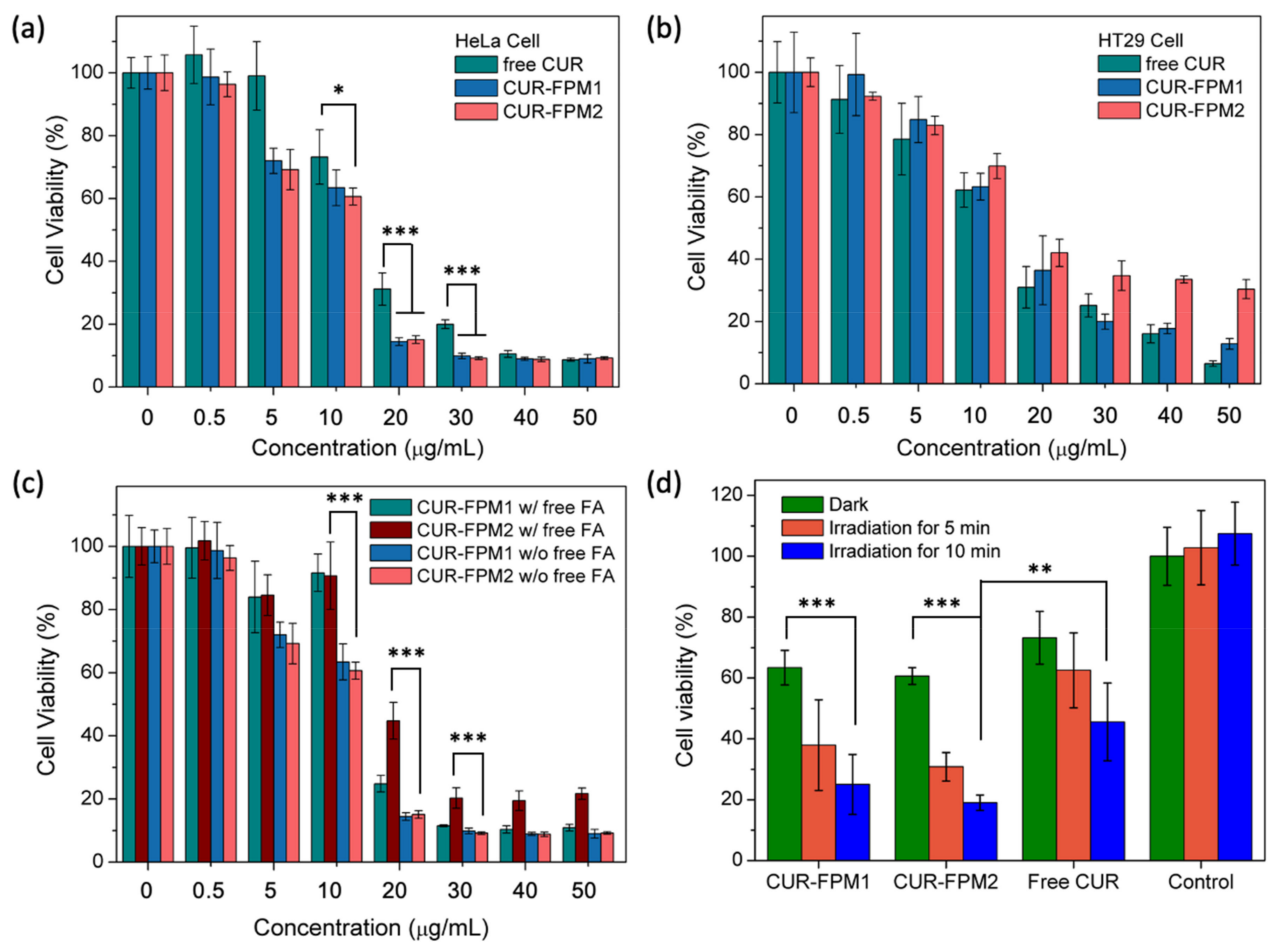
Cell viability of (**a**) HeLa cells and (**b**) HT-29 cells treated with free CUR, CUR-FPM1, and CUR-FPM2 micelles; (**c**) cell viability of HeLa cells treated with CUR-FPM1 and CUR-FPM2 micelles with or without the addition of 1 mM of free folic acid for competition study; (**d**) cell viability of HeLa cells treated with free CUR, CUR-FPM1, and CUR-FPM2 micelles for different irradiation times. The CUR concentration is fixed at 10 μg/mL. Data are presented as the mean ± standard deviation. *p* values: * *p* < 0.05, ** *p* <0.01, *** *p* < 0.001, significance level.

To evaluate the therapeutic effect and targeting characteristics of CUR-FPM micelles, CUR-FPM1, CUR-FPM2 micelles, and free CUR with a CUR concentration in the range of 0.5–50 μg/mL were tested against two different cancer cell lines, HeLa and HT-29 cells, because of their difference in the amount of folate receptor overexpression [33,34]. HeLa cells have more folate receptors overexpressed on the cell surface compared to that of HT-29 cells. MTT assays were used to determine the relative cell viability of HeLa or HT-29 cells. The viabilities of cells treated with blank micelles in the concentration range of 0.1 to 1.0 mg/mL for 24 h showed that the micelles were nontoxic (cell viability > 85%) and could be safely used as carriers for intracellular drug delivery (Figure S7). The half-maximal inhibitory concentration (IC_50_) values of CUR-FPM1, CUR-FPM2, and free CUR to HeLa cells were found to be 12.7, 12.3, and 15.5 μg/mL, respectively (Figure 4a), and to HT 29 cells were found to be 21.1, 18.8, and 13.9 μg/mL, respectively (Figure 4b). The lower IC_50_ for CUR-FPM micelles against HeLa cells was observed compared to the IC_50_ of CUR-FPM micelles against HT 29 cells. However, the IC_50_ for free CUR against both cell lines showed no obvious difference. This suggested the selective internalization of FA-conjugated micelles through the receptor-mediated endocytosis pathway caused by the overexpressed folate receptors on the surface of HeLa cells. The high-affinity binding between CUR-FPM micelles and HeLa cells increased their cellular internalization and significantly enhanced the cytotoxicity of HeLa. It was observed that HeLa cells treated with both CUR-FPM micelles had a similar cytotoxicity, though they had different folate contents. This indicated that both FA-conjugated micelles had a sufficient affinity to the folate receptor-expressing cancer cells. Meanwhile, it was found that HT 29 cells treated with CUR-FPM micelles had a lower cytotoxicity compared to free CUR, which might be due to there being less internalization of the micelles and a slow release of CUR.

On the other hand, a competition study against HeLa cells in the presence of excessive free folic acid (1 mM) was conducted simultaneously with CUR-FPM micelles incubated for 24 h. As shown in Figure 4c, with the competition-blocking of free folic acid, higher IC_50_ values (16.2 μg/ mL for CUR-FPM1 and 21.3 μg/mL for CUR-FPM2) were observed, implying the free folic acid blocked the specific folate-receptor mediate uptake and hindered the internalization of CUR-FPM micelles into HeLa cells. The IC_50_ of CUR-FPM2 was higher than that of CUR-FPM1. The large size of CUR-FPM2 micelles might inhibit the internalization of micelles via non-specific endocytosis. Taking all these results together, the selective internalization of folate-conjugated FPM micelles is promising for application in targeted drug delivery.

To evaluate the phototoxic potential of CUR-FPM micelles and the cytotoxicity of photoactive CUR on HeLa cells, CUR-FPM micelles and free CUR were investigated at an equivalent CUR concentration of 10 μg/mL in dark or light irradiation conditions. A control group without treatment of any micelle or drug showed no marked cytotoxic effect on HeLa cells under the same irradiation, indicating that the light dose used was not harmful to cells. Figure 4d shows that around 60% to 70% cell survival was observed when incubated with CUR-FPM or free CUR without light irradiation. This result indicated that the CUR released from FPM micelles resulted in the inhibition of cell growth. However, when cells were treated with PDT, the cell viabilities were further decreased along with increasing irradiation time. An enhanced cell death in CUR-FPM micelles compared to free CUR under light irradiation indicated that the CUR-FPM micelles had a high PDT efficacy and a good in vitro anti-cancer activity. The superior phototoxicity of CUR-FPM2 micelles in comparison to CUR-FPM1 micelles might be due to the greater folate conjugation of FPM2 and more efficient ROS production. The result was consistent with the intracellular ROS production.

### 3.6. Subcellular Localization and Cellular Uptake

**Figure 5:**
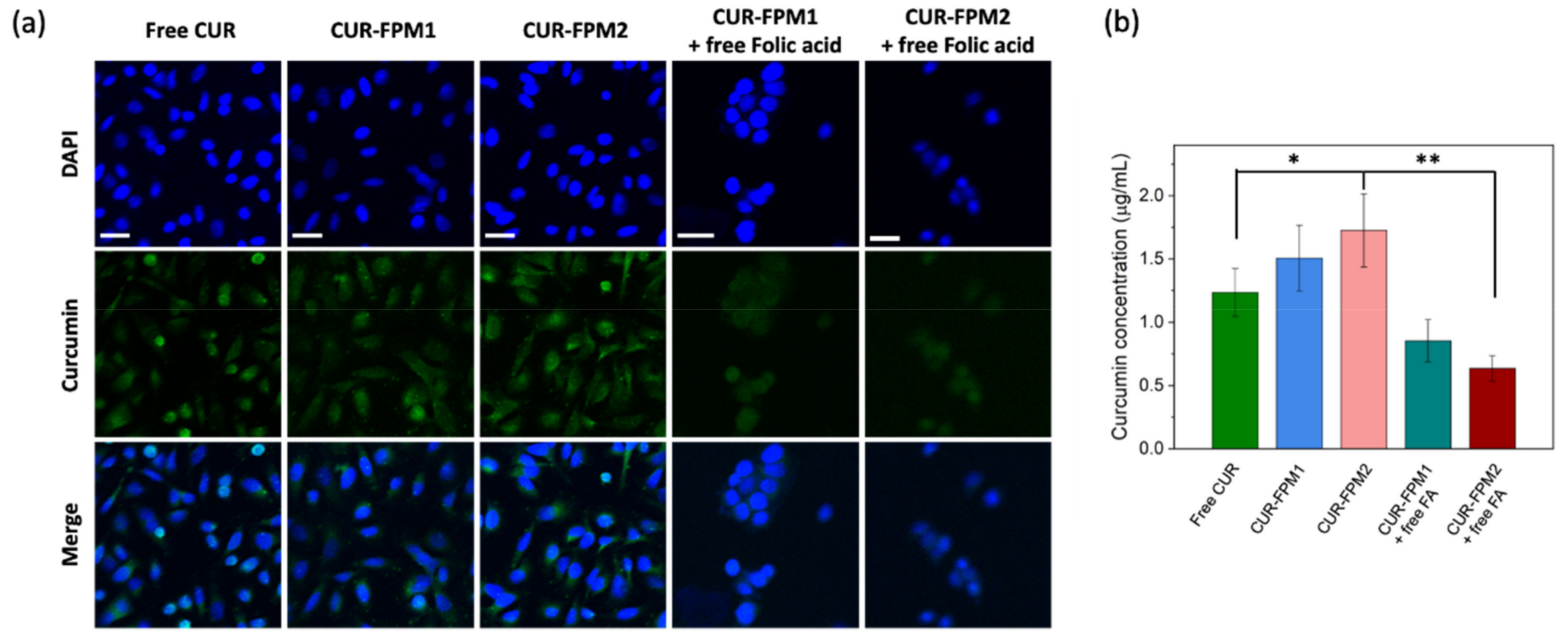
(**a**) CLSM images of HeLa cells treated with free CUR, CUR-FPM1, and CUR-FPM2 micelles with or without the addition of 1 mM of free folic acid for 2 h. Scale bar: 30 μm; (**b**) quantitation of curcumin in HeLa cells treated with free CUR, CUR-FPM1, and CUR-FPM2 micelles with or without the addition of 1 mM of free folic acid for 2 h. Data are presented as the mean ± standard deviation. *p* values: * *p* < 0.05, ** *p* < 0.01, significance level.

The subcellular localizations of CUR-FPM micelles were investigated by the inherent fluorescence of curcumin via confocal laser scanning microscopy. As shown in Figure 5a, green fluorescence was apparent in the cytoplasm and nucleus after 2 h of incubation with free CUR and CUR-FPM micelles. The fact that less CUR fluorescence of CUR-FPM micelles accumulated in the nucleus than that of free CUR might be attributed to the incomplete release of CUR from micelles over this time frame. When the cells incubated with free folic acid and CUR-FPM micelles, a much weaker fluorescence was observed. This result confirmed the inhibited cellular uptake of CUR-FPM micelles due to the free FA blocking to the folate receptors on the cell surface. The quantitative uptake of CUR in HeLa cells was further measured and is shown in Figure 5b. The amounts of curcumin in the CUR-FPM micelles-treated cells were higher compared to those of free CUR or CUR-FPM micelles with the addition of free FA. This was consistent with the results of their cytotoxicities and CLSM images. The enhanced cellular uptake of CUR-FPM micelles was due to the receptor-mediated endocytosis mechanism, whereas the free CUR internalized into cells was due to the passive diffusion mechanism [35]. The results also illustrated the CUR-FPM micelles designed in this study could provide an efficient route for delivering the drugs into cells through receptor-mediated endocytosis.

## 4. Conclusions

In summary, we prepared a series of new folate-conjugated FPM micelle systems to encapsulate CUR, which was used as an anticancer drug and photosensitizer for enhancing the anticancer efficiency and cell targeting. The FPM micelles significantly improved the CUR aqueous stability and had an appropriate micellar size and higher drug loading (6.3 ± 1.2%). A rapid CUR release to a total of approximately 85% was obtained in an acidic condition in comparison to about 66% in a neutral condition. In vitro cell viability tests confirmed that the CUR-FPM micelles induced a higher cytotoxicity of HeLa cells than free CUR did. The target specificity was evaluated using HeLa and HT 29 cells, showing a lower IC_50_ value (12.7 and 12.3 μg/mL for CUR-FPM1 and CUR-FPM2, respectively) toward HeLa cells, demonstrating the selective internalization of CUR-FPM micelles through the receptor-mediated endocytosis pathway. The competition study also confirmed that free folic acid blocked the interaction between the folate-conjugated micelles and FA receptor. Moreover, the CUR-FPM micelles displayed a superior PDT effect compared to free CUR in a light-dose dependent manner. All of these results indicate that the CUR-FPM micelle system is a promising platform for chemotherapy and CUR-mediated photodynamic therapy against folate receptor-overexpressing cancer cells.

## Figures and Tables

**Table 1:** Characterizations of the FPM micelles.

Micelles	FA conc. [g/g]		CMC ^c)^	Size (PDI) ^d)^	Zeta Potential ^d)^	Size ^e)^	LC/EE ^f)^
	Theo.^a)^	Exp.^b)^	[mg/mL]	[nm]	[mV]	[nm]	[%]
FPM1	0.034	0.012	2.5 × 10^−3^	162.7 ± 11.4 (0.24 ± 0.05)	−6.95 ± 2.85	159 ± 40	-
CUR-FPM1	0.034	0.012	-	216.8 ± 4.8 (0.15 ± 0.09)	−5.32 ± 3.03	225 ± 32	6.3 ± 1.2/52.3 ± 9.9
FPM2	0.025	0.022	6.0 × 10^−4^	192.7 ± 14.5 (0.12 ± 0.02)	−13.11 ± 11.58	172 ± 40	-
CUR-FPM2	0.025	0.022	-	237.6 ± 31.7 (0.13 ± 0.05)	−10.6 ± 2.61	238 ± 44	5.7 ± 0.7/52.3 ± 9.9

^a)^ Calculated with the chemical structure of FPM copolymer; ^b)^ determined by UV–visible spectrometer with folate calibration curve; ^c)^ determined by fluorescence probe technique using pyrene; ^d)^ determined by DLS measurement; ^e)^ obtained by TEM; ^f)^ the drug loading capacity (LC) and encapsulation efficiency determined using the UV–visible spectra with reference to a calibration curve of CUR.

## Supplementary Materials

The following are available online at https://www.mdpi.com/2073-4360/12/10/2280/s1: Scheme S1: Synthesis of propargyl folate; Figure S1: ^1^H NMR spectra of (a) PCL-Br and (b) PMPC-*b*-PCL (PM); Figure S2: ^1^H NMR spectrum of FA-PMPC-*b*-PCL (FPM); Figure S3: FTIR spectra of (a) N_3_-PM copolymer; (b) FPM copolymer and propargyl folate; Figure S4: TEM images of FPM1 and FPM2 micelles; Figure S5: Loading capacity and encapsulation efficiency of CUR in FPM micelles with different drug to polymer ratios; Figure S6: Calibration curve for curcumin in mixed solvent (DMSO: MeOH: THF = 1: 1: 1, v/v/v); Figure S7: Cell viability of HeLa cells and HT29 cells treated with PM micelles; Table S1: Characterization of PCL-Br and N_3_-PMPC-*b*-PCL (PM).
