# Supplementary material for: Folate-Targeted Curcumin-Encapsulated Micellar Nanosystem for Chemotherapy and Curcumin-Mediated Photodynamic Therapy
Source: Polymers (Basel). 2020 Oct 4;12(10):2280. doi: 10.3390/polym12102280 (PMC7599867; doi:10.3390/polym12102280)
Supplement: Supplementary file 1 [file polymers-12-02280-s001.pdf]

Supplementary Materials

Folate-Targeted Curcumin-Encapsulated Micellar Nanosystem for Chemotherapy and CUR-Mediated Photodynamic Therapy

*Yun Hsuan Lin and Ching-Yi Chen**

1. Materials

1-Ethyl-3-(3-dimethylaminopropyl)carbodiimide (EDC, Sigma-Aldrich), *N*-hydroxy-succinimide (Alfa Aesar), propargylamine (Alfa Aesar), sodium azide (99%, Panreac), sodium hydroxide (Showa) were used as received.

2. Synthesis of propargyl folate

Folic acid (1.0 g, 2.20 mmol) and *N*-hydroxysuccinimide (261 mg, 2.5 mmol) were dissolved in DMF (15 mL) and cooled in a ice water bath. Then, EDC (565 mg, 2.95 mmol) was added and the resulting mixture was stirred in a ice water bath for 3 h. Under nitrogen atmosphere, a solution of propargylamine (124.0 mg, 2.25 mmol) was added to the mixture and reacted at room temperature for 12 h. The product was obtained by precipitating the reaction mixture into DI water, followed by filteration and wahed with acetone. Then, dry under vaccum overnight to yield yellow-orange product around 1.0 g (92% yield). ^{1}H NMR (DMSO-d_6, ppm): δ 1.89 (m, 1H, glutamyl -CHCH_2), 2.04 (m, 1H, glutamyl -CHCH_2), 2.18 (m, 2H, glutamyl -CH_2CO), 3.06 (s, 1H, propargyl -CC*H*), 3.82 (m, 2H, propargyl -CH_2CCH), 4.35 (m, 1H, glutamyl -C*H*), 4.48 (d, 2H, pteroyl -CH_2NH), 6.63 (d, 2H, 2 × pteroyl -Ar*H*), 6.92 (br, 1 H, pteroyl CH_2N*H*), 7.65 (d, 2H, 2 × pteroyl -Ar*H*), 8.16 (d, 1H, glutamyl -N*H*CH), 8.29 (t, 1H, -N*H*CH_2CCH), 8.64 (s, 1H, pteroyl -NC*H*).

3. Hydrolysis of N_3-PMPC-*b*-PCL

The mixture of 80 mg N_3-PMPC-b-PCL and 3 mL 3% $NaOH_{(aq)}$/MeOH (3:2, v/v) solution was placed in a 10 mL round bottomflask and refluxed at 65 °C oil bath for 3 days. Then, the reaction mixture was dialyzed (MWCO 2000) against DI water for 1 day and MeOH for another day. The medium was replaced every 6 h. Finally, the product was precipitated in THF and dried in vacuum oven. The ^{1}H-NMR spectra of both copolymers showed the completely disappearance of the proton signals of PCL. The molecular weight and polydispersity index (PDI) were measured by gel-permeation chromatography (GPC) in 0.1 M $NaNO_{3(aq)}$ mobile phase relative to polyethylene glycol.

4. General characterization

^{1}H NMR spectra were measured in $CDCl_3$, DMSO-d_6 or $CDCl_3$/CD_3OD cosolvent by using a Bruker-DPX-400 instrument spectrometer operating at 400 MHz. The molecular weights and polydispersity of the PCL-Br were determined by GPC equipped with Waters 1513 Isoscratic HPLC pump and a refractive index (Waters 2410) detector. The measurements were taken at 40°C with THF as the eluent (1 mL/min) and calibrated with PS standards in the range of absolute molecular weight from 580 to 371 kg/mol. For the hydrolyzed N_3-PMPC, their molecular weights and polydispersity were determined by GPC (Schambeck SFD Gmbh, Germany) with RI 2000 detector with 0.1 M $NaNO_{3(aq)}$ as the eluent (0.5 mL/min) at 35 °C. Polyethylene glycol in the range of absolute molecular weight from 4K to 70K were used as calibration standards.

5. Evaluation of critical micelle concentration (CMC)

The CMC of the block copolymers in water was determined by a fluorescence probe technique using pyrene as a hydrophobic fluorescent probe. The polymer solution with a concentration of 1.0×10^{-6} to 0.5 mg/mL was added into the vials containing pyrene (6.6×10^{-7} M). These solutions were stirred at room temperature for 24 h before measurements. Emission spectra of pyrene molecules were excited at 335 nm using a fluorescence spectrometer (Hitachi,

F-7000). The intensity ratios of I_3 at 384 nm to I_1 at 373 nm were analyzed as a function of block copolymer concentration to determine the CMC of block copolymers.

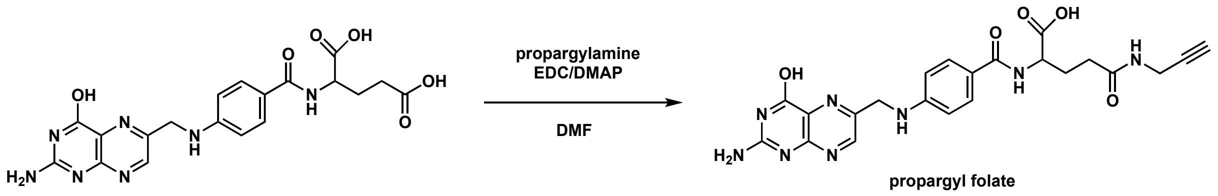

Scheme S1. Synthesis of propargyl folate.

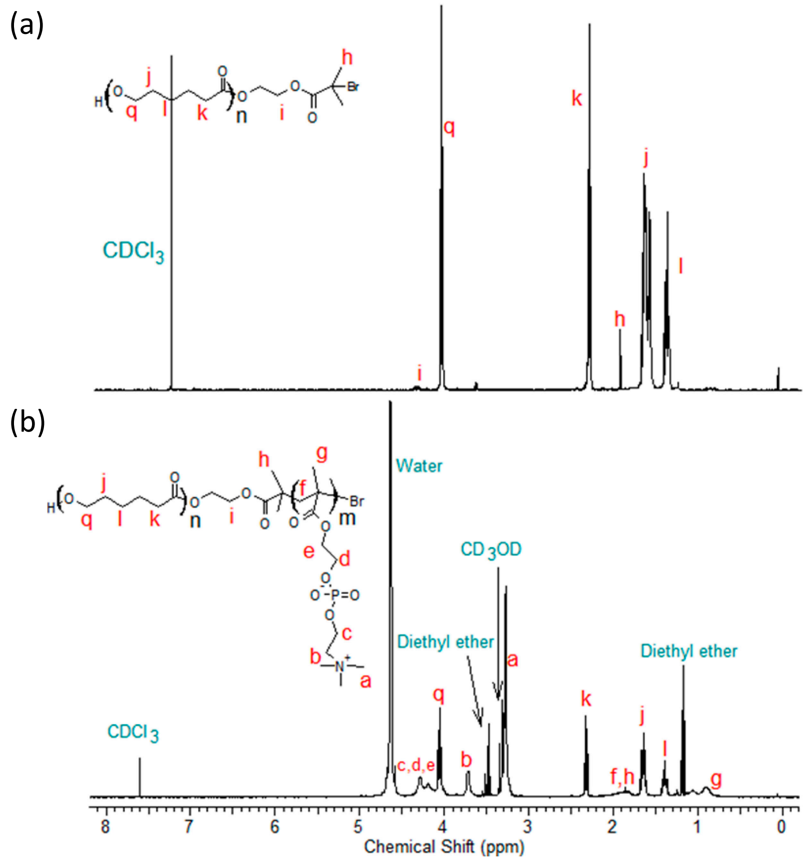

Figure S1. ^{1}H NMR spectra of (a) PCL-Br in $CDCl_3$ and (b) PMPC-*b*-PCL (PM) in $CDCl_3/CD_3OD$ mixture (1: 1, v/v).

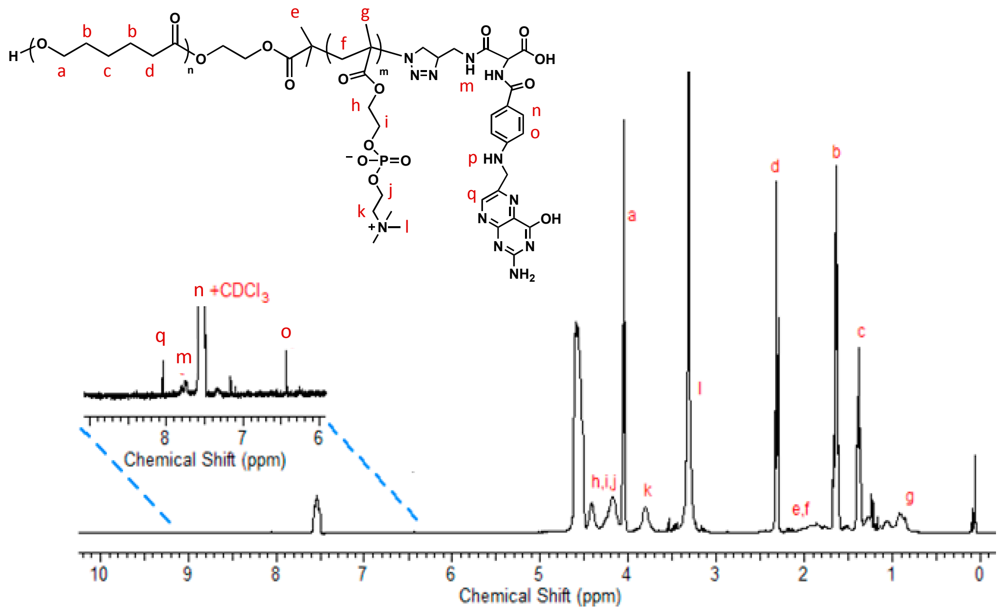

Figure S2. ^{1}H NMR spectrum of FA-PMPC-*b*-PCL (FPM) in CDCl3/CD3OD mixture (1: 1, v/v).

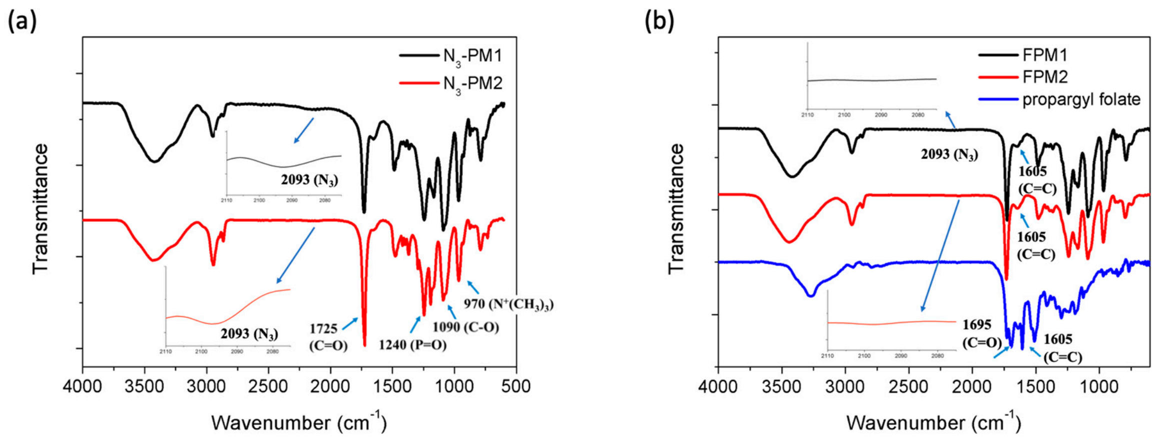

Figure S3. FTIR spectra of (a) N3-PM copolymer and (b) FPM copolymer and propargyl folate.

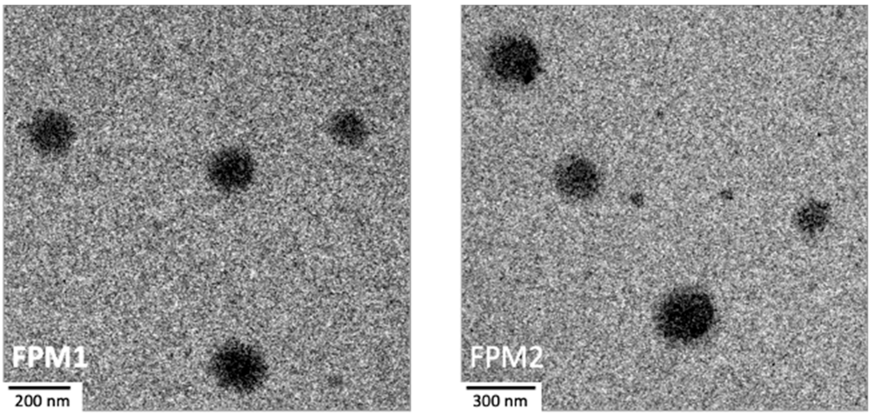

Figure S4. TEM images of FPM1 and FPM2 micelles

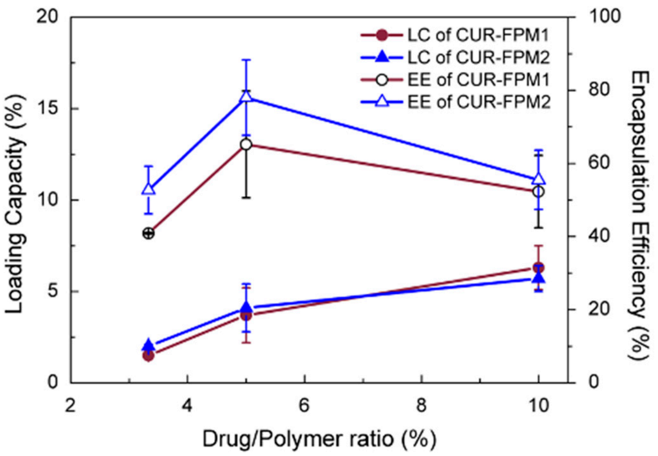

Figure S5. Loading capacity and encapsulation efficiency of CUR in FPM micelles with different drug to polymer ratios.

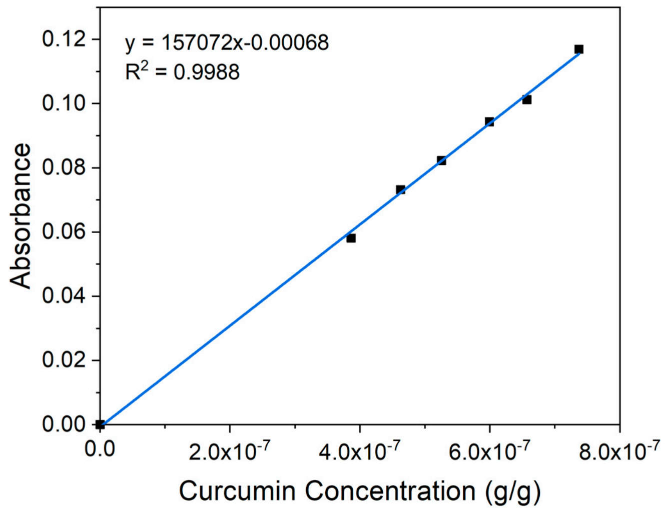

Figure S6. Calibration curve for curcumin in mixed solvent (DMSO: MeOH: THF = 1: 1: 1, v/v/v)

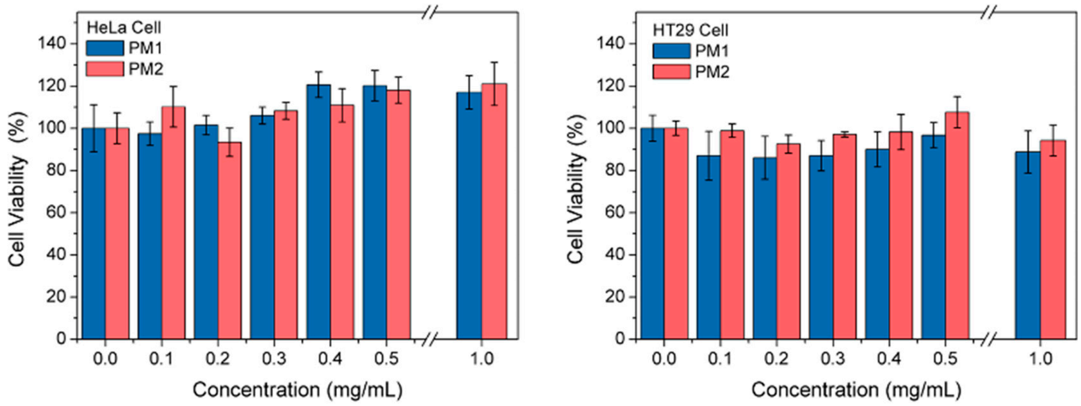

Figure S7. Cell viability of HeLa cells and HT29 cells treated with PM micelles.

Table S1. Characterization of PCL-Br and N_3-PMPC-*b*-PCL (PM)

Polymer	DP [a)]		$M_{n\,(NMR)}$ (Da) [a)]	$M_{n\,(GPC)}$	
	CL	MPC		PCL-Br Da (PDI)[b)]	N_3-PMPC Da (PDI)[b)]
N_3-PM1	30	35	13965	7600 (1.25)	16400 (1.53)
N_3-PM2	80	32	18779	13200 (1.30)	16300 (1.67)

[a)] Degree of polymerization (DP) and number-average molecular weight (M_n) were analyzed from ^{1}H-NMR spectra; [b)] Number-average molecular weight and polydispersity index (PDI) were determined by GPC in THF mobile phase for PCL-Br, and 0.1 M $NaNO_3$ mobile phase for hydrolyzed N_3-PMPC.
